# On the Use of the Thymus Gland

**Published:** 1845-03

**Authors:** 


*On the use of the Thymus Gland.*—Dr. Picci, after glancing at the theories of his predecessors, suggests that the use of this gland is chiefly of a mechanical nature; viz. to occupy a certain space within the thoracic cavity, while the lungs remain unexpanded in the fœtus; and thus to prevent the ribs and sternum from falling in too much upon these vital organs. The size of the thymus is inversely as the volume of the lungs; and, when the latter become dilated after birth by the admission of air into their cells, the former immediately begins to shrink and become atrophied. In truth, it is only in the adult that the thoracic parieties are moulded completely upon the lungs; for, in infancy and youth, it is rather the thymus gland that is, in their place, moulded upon the thorax.

The situation of this gland in the interior mediastinum and along the median line, the very nature of its tissue, and the greater expansion and developement of its inferior half, are adduced as arguments in favor of the opinion now adduced. Besides the well-known circumstance that, in those new born children in whom the thorax is very largely developed, the thymus continues to increase gradually even to the end of the second year, it deserves notice that all those animals, in which the lungs are similar to those in the human subject, are provided with this gland; whereas, we find it to be entirely wanting in those which breath by branchiæ or membranous lungs. In hybernating animals, also, the thymus exhibits alternations of enlargement and decrease, according to the state of the respiratory organs. In the Amphibia it attains its maximum of development.

The circumstance, too, of the gland being usually rather larger than ordinary in phthisical patients may be mentioned as lending some probability to the view we have proposed.—*Ibid, from Annali Universali.*

